# Serotonin receptors and their association with the immune system in the gastrointestinal tract of weaning piglets

**DOI:** 10.1186/s40813-022-00250-5

**Published:** 2022-01-28

**Authors:** Lluís Fabà, Nienke de Groot, Guillermo Ramis, Carolina G. Cabrera-Gómez, John Doelman

**Affiliations:** 1 Trouw Nutrition R&D, Boxmeer, The Netherlands; 2 Trouw Nutrition Innovation, Amersfoort, The Netherlands; 3 Dpto. Producción Animal, Facultad de Veterinaria, Universidad de Murcia, Murcia, Spain

**Keywords:** Serotonin, 5-HT, Immunity, Inflammation, Piglet, Weaning, Gut health

## Abstract

**Background:**

Immune cell activation and perpetuation of inflammation have been attributed to the neurotransmitter serotonin (5-hydroxytryptamine; 5-HT). Our hypothesis was that the 5-HT system plays a role in GI health and immunity in post-weaning piglets. A disruption of the 5-HT system post-weaning with transcriptional upregulation of 5-HT receptors may be linked to increased cytokine mRNA abundance and immune system activation.

**Methods:**

The objective of this exploratory study was to assess the relationship between 5-HT receptor expression and immune system biomarkers in piglets at 1 (n = 9) and 15 (n = 10) days post-weaning. The mRNA transcript abundance of three 5-HT receptors (5-HTR3, 5-HTR4, and 5-HTR7) measured in jejunum and colon tissues were used to determine the relationship with the immune system and jejunal morphometry at 2 timepoints post-weaning using correlations, mixed models, and multivariate analysis techniques.

**Results:**

Overall, 5-HT receptor mRNA expression decreased from day 1 to day 15 post-weaning. Time × tissue interactions showed the lowest 5-HTR3 expression in the colon and lower 5-HTR7 expression in the jejunum at 15 days post-weaning. 5-HTR3 and 5-HTR4 expression were negatively associated with pro-inflammatory (IFN-ɣ) and anti-inflammatory (IL-10 and IL-12β) cytokines in jejunum, and with TNF-α in the colon at 1-day post-weaning. At 15 days post-weaning, 5-HTR3 in the colon was negatively associated with pro-inflammatory (IL-1α, IL-1β, TNF-α, IL-8, and IFN-ɣ) and anti-inflammatory (IL-10 and IL-12β) cytokines. Furthermore, 5-HTR7 expressed a predominantly pro-inflammatory profile (IFN-α, IL-1α, IL-1β, IL-8, TNF-α and IL-12α) in the jejunum at the same timepoint, whereas colonic 5-HTR7 expression was negatively correlated with IL-1α, IL-1β, IL-10 and TGF-β. Lastly, positive correlations were found for increased expression of 5-HTR4 receptor with villus height, 5-HTR7 receptor expression and crypt depth, and increased expression of 5-HTR3 and 5-HTR4 receptor with villus height to crypt depth ratio at 1-day post-weaning.

**Conclusions:**

The 5-HT receptor mRNA abundance was associated with the immune system and intestinal morphometry in piglets. The 5-HT receptors were highly expressed at weaning in both jejunum and colon tissues relative to 15 days post-weaning. Although a clear relationship between immune system and 5-HTR expression is observed, particularly at day 15, a cause-consequence cannot be proven with current data. Further research is warranted to elucidate the effects of 5-HT on gastrointestinal inflammation during the weaning process in piglets, which could be the basis for new interventions to ease weaning stress.

## Background

Serotonin (5-hydroxytryptamine; 5-HT) is a monoamine neurotransmitter predominantly synthesized by enterochromaffin cells (ECs) in the gastrointestinal (GI) tract and by neurons of the central and enteric nervous systems (CNS and ENS) to participate in sensory-motor and secretory functions [1]. Recently, a more complete immunological function in the gut has been described, as reviewed by Banskota et al. [2], which includes roles in immune cell activation and perpetuation of the inflammatory response. These roles are highly relevant for human medicine and may have implications in companion animals and livestock. In pigs, elucidation of 5-HT functionality could improve the understanding of the GI tract immune system during key production phases such as weaning. Furthermore, the integrated 5-HT pathway and functional intestinal mucosa presents a novel innovation target for the development of preventative and therapeutic technologies.

The 5-HT system operates through seven receptor families, of which five (5-HTR1, 5-HTR2, 5-HTR3, 5-HTR4 and 5-HTR7) are expressed in the GI tract within smooth muscle, enteric neurons, enterocytes, and immune cells [3–5]. In terms of functionality, fluid and mucus secretion and ion transport are regulated by epithelial 5-HTR2 receptors and neuronal 5-HTR1, 5-HTR3 and 5-HTR4 receptors [6]. Antagonists for 5-HTR3 inhibit basal levels of chloride and water secretion, whereas agonists for 5-HTR4 stimulate them [7]. Roles of 5-HT on intestinal motility are also well described; for example, agonists of 5-HTR3 and 5-HTR4 elevate peristalsis whereas the use of antagonists inhibit it [8]. Beyond sensory-motor and secretory functions, 5-HT signaling increases morphometric and proliferative parameters in the small intestine and improves absorptive function [9] and modulate immune functions [2]. In fact, most human and rodent immune cells express 5-HT receptors [3, 10]. The recruitment of innate immune cells at the site of inflammation is co-regulated by 5-HT [5, 11]. 5-HT is reported to enhance the activity of natural killer cells (NK) via 5-HTR7 [4], influencing cell activation and proliferation. In dendritic cells (DC), 5-HT increases pro-inflammatory T cell response [12].

Human patients with inflammatory bowel disease (IBD) have increased levels of 5-HT in the GI tract [13, 14]. Using a murine colitis model induced with 2,4-dinitro-benzenesulfonic acid (DNBS) [15] or dextran sodium sulfate (DSS) [16], a similar relationship has been described. The enzyme tryptophan hydroxylase 1 (Tph1) is needed for 5-HT production in EC. Interestingly, using DSS- and DNBS-inflammation models, the severity of colitis was reduced in Tph1 knockout mice, which was linked to lowered capability for producing 5-HT in the gut [17]. Similarly, the work of Müller et al. [11] indicates a role 5-HT in the inflammation process, reporting that 5-HT binding to 5-HTR3, 5-HTR4 and 5-HTR7 receptors up-regulated the production of the pro-inflammatory cytokine IL-6. Furthermore, the authors report that 5-HT induced the maturation of DCs, resulting in a phenotype of increased IL-10 and decreased IL-12p70 secretion.

Published data on the role of 5-HT in swine is largely based on 5-HT cognitive function, pertaining to welfare and behavior, and linked to the essential amino acid tryptophan [18–20]. However, little is known of its role in the swine GI tract during periods of stress and inflammation. Carlson et al. [21, 22] describe a post-weaning 5-HT-induced chloride secretion pattern in intestinal tissue ex vivo. They report that the secretory response to 5-HT was more sensitive immediately following weaning compared to 15 days later, suggesting that this could be a risk factor for diarrhea. If increased 5-HT receptor sensitivity at weaning increases secretory function, it could be hypothesized that 5-HT-mediated immune signaling through cytokines may increase as well. More specifically, the 5-HT system could play a role in post-weaning inflammation and changes in villi morphometry that are influenced by the abrupt dietary change to solid feed, thereby influencing post-weaning nutrient absorption and growth performance in piglets.

Our hypothesis was that the 5-HT system plays a role in GI immunity in post-weaning piglets. A disruption of 5-HT system post-weaning with transcriptional upregulation of 5-HT receptors may be linked to increased cytokine mRNA abundance as well as other immune system biomarkers. Here, we describe the gene expression of three 5-HT receptors, selected as proof-of concept and being the most associated with GI tract and immunity [3–11], in jejunal and colonic tissues and their relationship with immune system biomarkers and intestinal morphometry (jejunum) at 1- and 15-days post-weaning.

## Results

All pigs were necropsied for evaluation of gross lesions, but no pathological findings were observed. Piglets sampled at day 15 post-weaning had an average daily gain (ADG) of 265 g/d, an average daily feed intake of 255 g/d, and a calculated feed efficiency (FE, gain/feed intake) of 1.04. The FE > 1 is obviously not a correct estimation of actual growth efficiency but is a common consequence of subclinical oedema in piglets early post-weaning.

### Mixed models for time and tissue effects

Using the MIXED procedure analysis for gene expression, significant effects of time (1-day post-weaning vs. 15 days post-weaning) and time × tissue (jejunum vs colon) interactions were identified (Table 1). mRNA abundance for 5-HTR3 decreased with time in both tissues, while relative expression was decreased in colon at 15 days post-weaning (*P* < 0.001). Similarly, 5-HTR7 expression decreased with time in both tissues, although relative abundance was lower in jejunum relative to colon tissue at 15 days post-weaning (*P* = 0.001). A decrease over time was also observed for 5-HTR4 transcript abundance (*P* < 0.001), but no tissue or time × tissue interaction was noted (*P* > 0.050; Table 1).

**Table 1:** Tissue^1^ serotonin receptor^2^ expression, immune biomarkers^3^, IgA-producing cell abundance^4^ and jejunum morphometry in piglets at 1- and 15-days post-weaning

	1-day post-weaning		15 days post-weaning		SE	*P* value		
	Jejunum	Colon	Jejunum	Colon		Time	Tissue	Time * Tissue
5-HTR3	21.9^a^	22.7^a^	15.2^b^	3.86^c^	0.484	< 0.0001	< 0.0001	< 0.0001
5-HTR4	3.37	2.76	1.52	2.06	0.294	< 0.0001	0.909	0.304
5-HTR7	3.07^a^	2.00^ab^	0.911^b^	2.46^a^	0.471	0.024	0.500	0.001
IL-1α	4.83	6.84	5.43	8.72	1.037	0.122	0.002	0.419
IL-1β	5.95	3.19	6.10	4.26	0.647	0.306	0.000	0.436
IL-6	2.33	3.01	2.36	3.57	0.354	0.384	0.008	0.438
IL-8	2.01	2.75	2.33	4.67	0.534	0.010	0.001	0.057
IL-10	3.14	1.56	3.98	3.44	0.352	0.001	0.002	0.110
IL-12α	7.98	4.81	7.29	4.93	0.846	0.711	0.001	0.595
IL-12β	4.74	3.57	5.13	3.23	0.433	0.944	0.001	0.307
TNF-α	12.8	22.3	12.5	20.6	1.386	0.387	< 0.0001	0.554
IFN-α	3.24	2.48	2.77	1.75	0.537	0.183	0.054	0.772
IFN-γ	3.39	2.67	5.03	3.29	0.766	0.011	0.006	0.218
TGF-β	12.9	11.4	13.0	11.5	0.230	0.650	< 0.0001	0.925
IgA-cells	12.3	8.51	14.7	11.3	1.545	0.082	0.017	0.890
Villus height	434		479		21.95	0.174		
Crypt depth	221		285		12.94	0.002		
Villus:crypt ratio	1.986		1.744		0.144	0.239		

^1^Jejunum and colon tissues from 1-day post-weaning piglets (n = 9) or 15 days post-weaning (post-weaning; n = 10)

^2^mRNA expression (log 2) of serotonin (5-HT) receptors (HTR3, HTR4, and HTR7)

^3^Cytokines = mRNA expression (log 2) of interleukin IL-1α, IL1-β, IL-6, IL-8, IL-10, IL-12p35 (IL-12α), IL-12p40 (IL12-β), tumor necrosis factor alpha (TNF-α), interferon alpha and gamma (IFN-α and IFN-γ), transforming growth factor betta (TGF-β)

^4^IgA-cells: counted in 10 non-overlapped field of 25.000 μm^2^

^a–c^Different superscripts within a row indicate a significant difference (*P* < 0.05)

Colonic IL-8 mRNA expression tended to be higher at 15 days relative to 1 day post-weaning (*P* = 0.057; Table 1). Interferon (IFN)-α expression tended to be lower in the colon compared to the jejunum, independent of time (*P* = 0.054). Tissue mRNA abundance of all other analyzed cytokines, including IFN-γ, IL-1β, IL-10, IL-12α, IL-12β, transforming growth factor (TGF)-β were higher, while IL-1α, IL-6, IL-8 and tumor necrosis factor (TNF)-α were lower in the jejunum compared to the colon (*P* < 0.010; Table 1) independently of time. Abundance of IFN-γ, IL-8 and IL-10 transcripts were significantly higher (*P* < 0.02) at 15 days relative to 1-day post-weaning independent of tissue. A higher abundance of IgA-producing cells was observed in the jejunum compared to the colon (*P* = 0.017; Table 1).

Jejunal crypt depth was increased at 15 days relative to 1-day post-weaning (*P* = 0.002), while no time effect for villus height or villus height to crypt depth ratio was observed (Table 1).

### Correlations

**Fig. 1:**
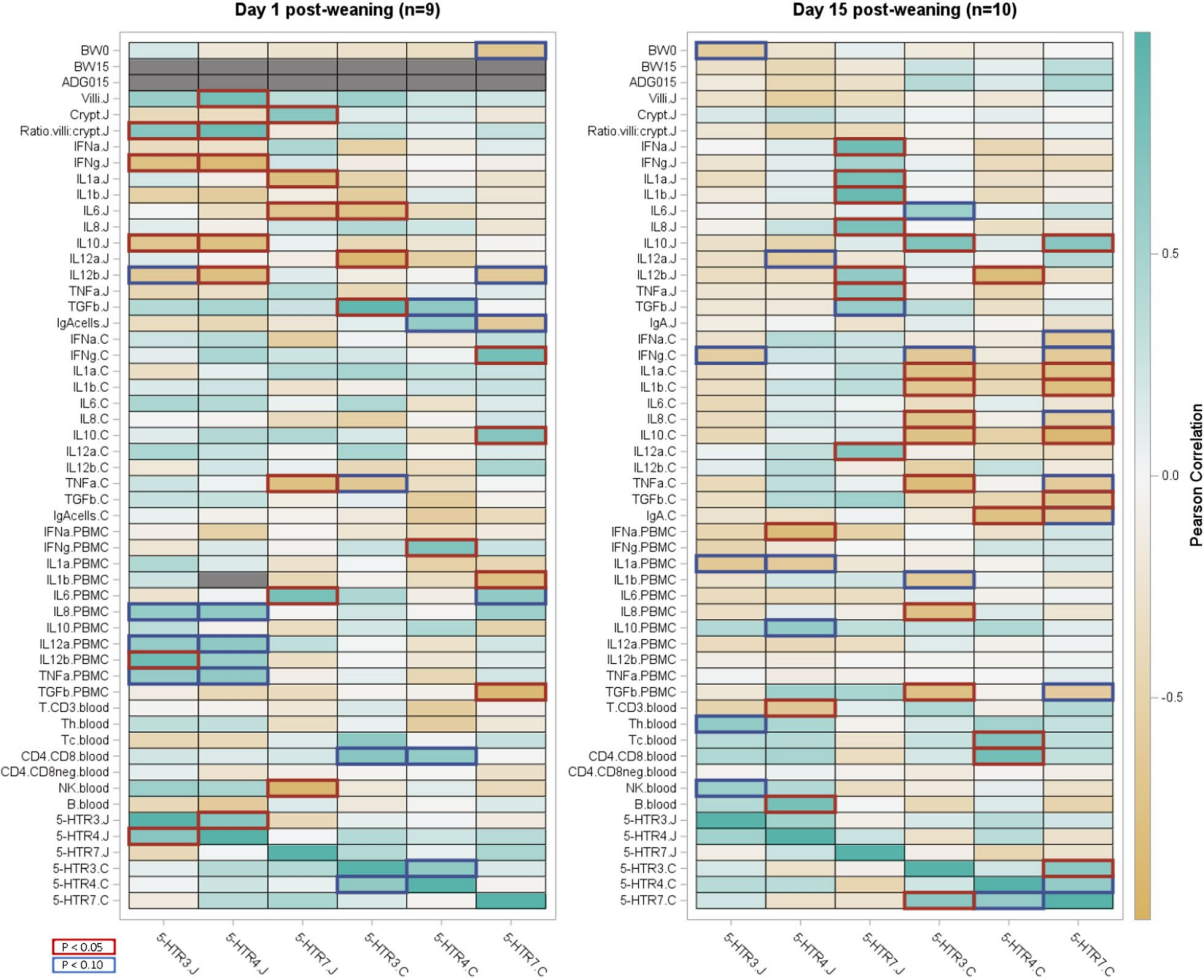
Heatmaps for Pearson r correlation^1^ between serotonin receptors^2^, performance traits^3^, jejunum morphometry^4^ and immune biomarkers^5^ in 1- and 15-day post-weaning piglets. ^1^Pearson r correlation^1^ with *P* values < 0.05 are outlined in red and *P* values < 0.10 are outlined in blue. ^2^mRNA expression (log 2) of serotonin (5-HT) receptors (5-HTR3, 5-HTR4, and 5-HTR7) for jejunum (J) and colon (C). ^3^Body weight (BW) at weaning (BW0), at 15 days post-weaning (BW15) and average daily gain between weaning and 15 days post-weaning (ADG015). ^4^Villi height, crypt depth and ratio villi height to crypt depth for jejunum. ^5^Immune parameters = mRNA expression (log 2) of 11 cytokines: interleukin (IL)-1α (IL1a), IL-1β (IL1b), IL-6 (IL6), IL-8 (IL8), IL-10 (IL10), IL-12p35 (IL12a), IL-12p40 (IL12b), tumor necrosis factor alpha (TNFa), interferon alpha and gamma (IFNa and IFNg), transforming growth factor betta (TGFb) for jejunum, colon and PBMCs; and IgA-producing cells (IgAcell) for jejunum (J) and colon (C); and lymphocytes B phenotype CD21+ (B), natural killer cells phenotype CD3−16+56+ (NK) and lymphocytes T phenotypes CD3+ (CD3), T helper CD3+CD4+ (Th), T cytotoxic CD3+CD8+ (Tc), CD4+CD8+ (CD4CD8), and CD4−CD8− (CD4CD8neg) from blood

Heatmaps depict the relative correlation coefficients between 5-HT receptor mRNA expression, BW, ADG, cytokine gene expression for each tissue, histomorphometry for the jejunum and the immune cell count (Fig. 1), with intensity of the correlations indicated by color grade (statistical significance or tendency outlined in red or blue, respectively). Amongst receptors, 5-HTR3 expression was elevated at day 1 relative to day 15 post-weaning and was positively correlated with 5-HTR4 expression at 1-day post-weaning in the jejunum (r = 0.70, *P* = 0.035), and tended to be in the colon (r = 0.64, *P* = 0.085). In colonic tissue and 15 days post-weaning, 5-HTR3 expression correlated positively with 5-HTR7 (r = 0.67, *P* = 0.034), while mRNA expression of each was relatively decreased and increased, respectively (Table 1). Decreased 5-HTR4 receptor transcript abundance at day 15 tended (r = 0.62, *P* = 0.057) to correlate positively with higher expressed 5-HTR7 in colon. There was a lack of correlation amongst 5-HT receptor gene expression values between the jejunum and colon (Fig. 1).

Analysis of the relationship between immune parameters and 5-HT receptors (6 × 42 correlation matrix) revealed 19 statistically significant correlations (*P* < 0.05) and a further 15 tendencies (*P* < 0.10) at day 1 post-weaning. Similarly, at 15 days post-weaning, 27 correlations were significant (*P* < 0.05) and 17 were identified as tendencies (*P* < 0.10) (Fig. 1). At 1-day post-weaning within tissue type, negative correlations were found between 5-HTR3 and 5-HTR4 receptor expression and IFN-ɣ (pro-inflammatory). Similarly, jejunal IL-10 and IL-12β (anti-inflammatory) expression were negatively correlated with 5-HTR3 and 5-HTR4, whereas colonic 5-HTR3 receptor expression was negatively associated with TNF-α. Colonic 5-HTR3 receptor expression, with lowest 5-HTR3 mRNA amounts, was negatively correlated with IL-1α, IL-1β, TNF-α, IFN-ɣ (pro-inflammatory) and IL-8 (pro-inflammatory chemokine), whereas negative correlations were found with IL-10 and IL-12β at day 15 post-weaning. The lowest abundance of 5-HTR3 mRNA, observed in the colon at 15 days post-weaning, was negatively correlated with IL-1α, IL-1β, TNF-α, IFN-ɣ, IL-8, IL-10, and IL-12β.

Furthermore, correlations for day 15 post-weaning 5-HTR7 receptor expression differed between tissues. Within the jejunum, 5-HTR7 receptor expression was lower (Table 1) and correlated positively with IFN-α, IL-1α, IL-1β, IL-8, TNF-α and IL-12α (Fig. 1). In contrast, colonic 5-HTR7 receptor expression was higher (Table 1) and correlated negatively with pro-inflammatory biomarkers IL-1α, IL-1β, and anti-inflammatory/immune inhibition biomarkers IL-10 and TGF-β (Fig. 1).

5-HTR4 receptor expression and villus height were positively correlated (r = 0.81, *P* = 0.008), as were 5-HTR7 receptor expression and crypt depth (r = 0.72, *P* = 0.04) at 1-day post-weaning. Similarly, 5-HTR3 (r = 0.70; *P* = 0.036) and 5-HTR4 (r = 0.86; *P* = 0.003) correlated positively with villus height to crypt depth ratio in the jejunum. Additionally, there were tendencies for negative correlations (*P* < 0.10) between 5-HT receptor expression and 1-day post-weaning BW, colon 5-HTR7 expression at day 1 (r =  − 0.66, *P* = 0.055), and with 5-HTR3 expression in the colon at 15 days post-weaning (r =  − 0.57, *P* = 0.084) (Fig. 1).

### Principal component analysis

**Fig. 2:**
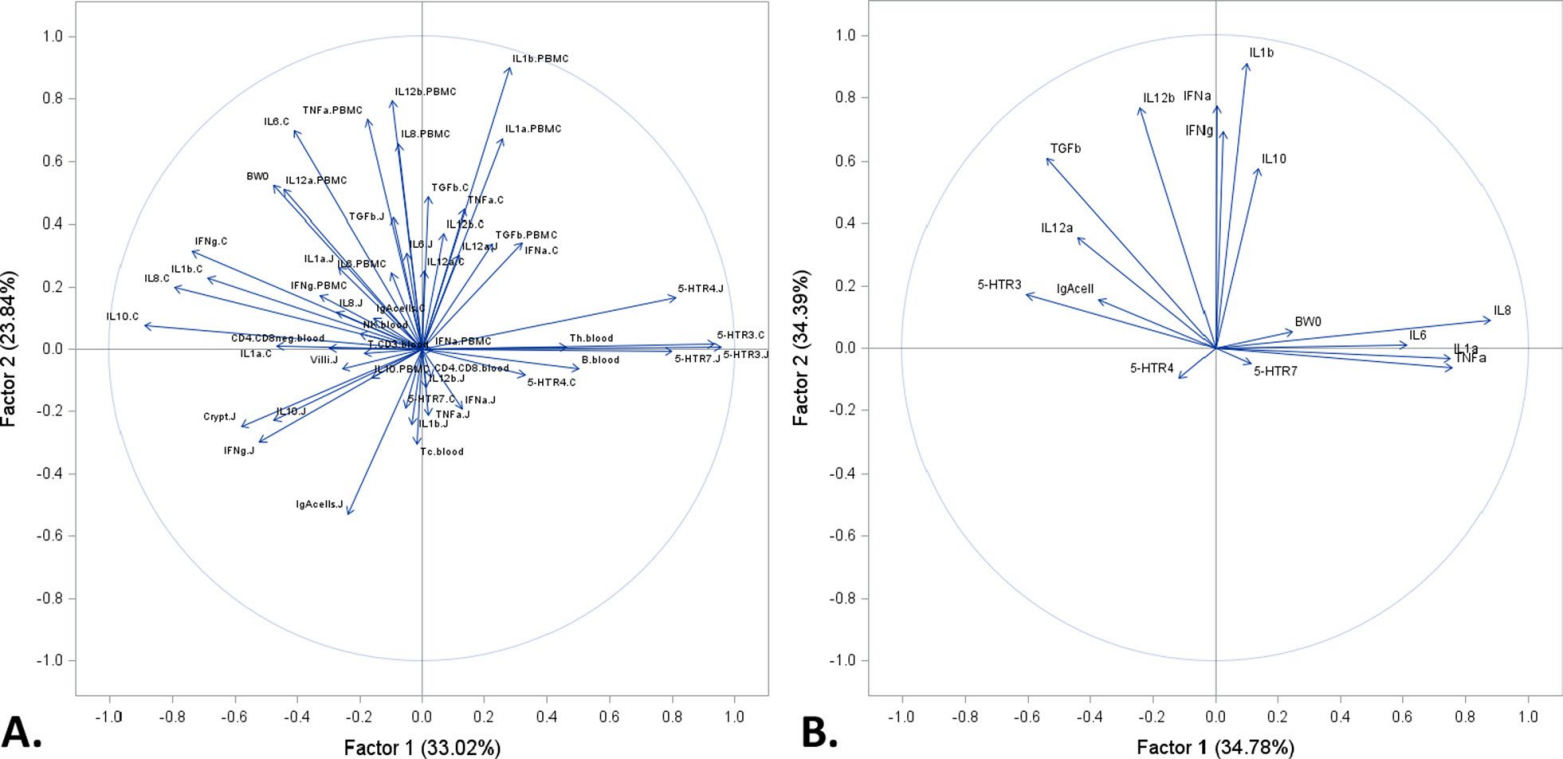
Principal component analysis for: **A** dataset-A, and **B** dataset-C. **A** Variance of serotonin receptors^1^, and 12 immune parameters^2^ coded as jejunum (J), colon (C) or peripheral blood mononuclear cells (PBMC), 7 immune cell types^3^, jejunum villus height and crypt depth, and weaning body weight (BW0). **B** Dataset-C—variance serotonin receptors^1^, and immune parameters^2^ (observation together regardless of tissue jejunum and colon). Data combines piglets at day 1 post-weaning (n = 9) and piglets at 15 days post-weaning (n = 10). ^1^ mRNA expression (log 2) of serotonin (5-HT) receptors (5-HTR3, 5-HTR4, and 5-HTR7) for jejunum and colon. ^2^mRNA expression (log 2) of 11 cytokines: interleukin IL-1α (IL1a), IL-1β (IL1b), IL-6 (IL6), IL-8 (IL8), IL-10 (IL10), IL-12p35 (IL12a), IL-12p40 (IL12b), tumor necrosis factor alpha (TNFa), interferon alpha and gamma (IFNa and IFNg), transforming growth factor betta (TGFb) for jejunum, colon and PBMCs; and IgA-producing cells (IgAcell) for jejunum and colon. ^3^Lymphocytes B phenotype CD21+ (B), natural killer cells phenotype CD3−16+56+ (NK) and lymphocytes T phenotypes CD3+ (CD3), T helper CD3+CD4+ (Th), T cytotoxic CD3+CD8+ (Tc), CD4+CD8+ (CD4CD8), and CD4−CD8− (CD4CD8neg)

Principle component analysis (PCA) of dataset-A (see “Statistical analysis” section) revealed a total variance of 57% explained by 2 factors of 33% and 24%, respectively (Fig. 2A). Based on the plot of scores and eigen vectors, Factor 1 (X axis), which explains 58% of the total variance, consisted of 5-HTR3, 5-HTR4 and 5-HTR7 receptor expression measured in the jejunum, 5-HTR3 from the colon, grouped together with lymphocyte T helper CD3+CD4+ (Th) and lymphocytes B. Principal Component Factor 2 (Y axis) explained 42% of the total variance, where 5-HTR receptor abundance appears more central in the scatter plot and the immune parameters, being more extreme, explain more of the variance. The PCA performed on dataset-C (Fig. 2B) identified 2 factors that explain 59% of the eigenvalue variance. The most opposing components in Factor 1 (X axis) are 5-HTR3 grouped with IL-12α, TGF-β and IgA-cells, contrasted against IL-8, IL-1α, TNF-α and IL-6. In Factor 2 (Y axis), receptors 5-HTR4 and 5-HTR7 grouped with weaning BW, IL-8, IL-1α, TNF-α and IL-6, contrasted against a group consisting of TGF-β, IFN-α, IFN-γ, IL-1β, IL-12β, and IL-10.

### Partial least squares regression

**Fig. 3:**
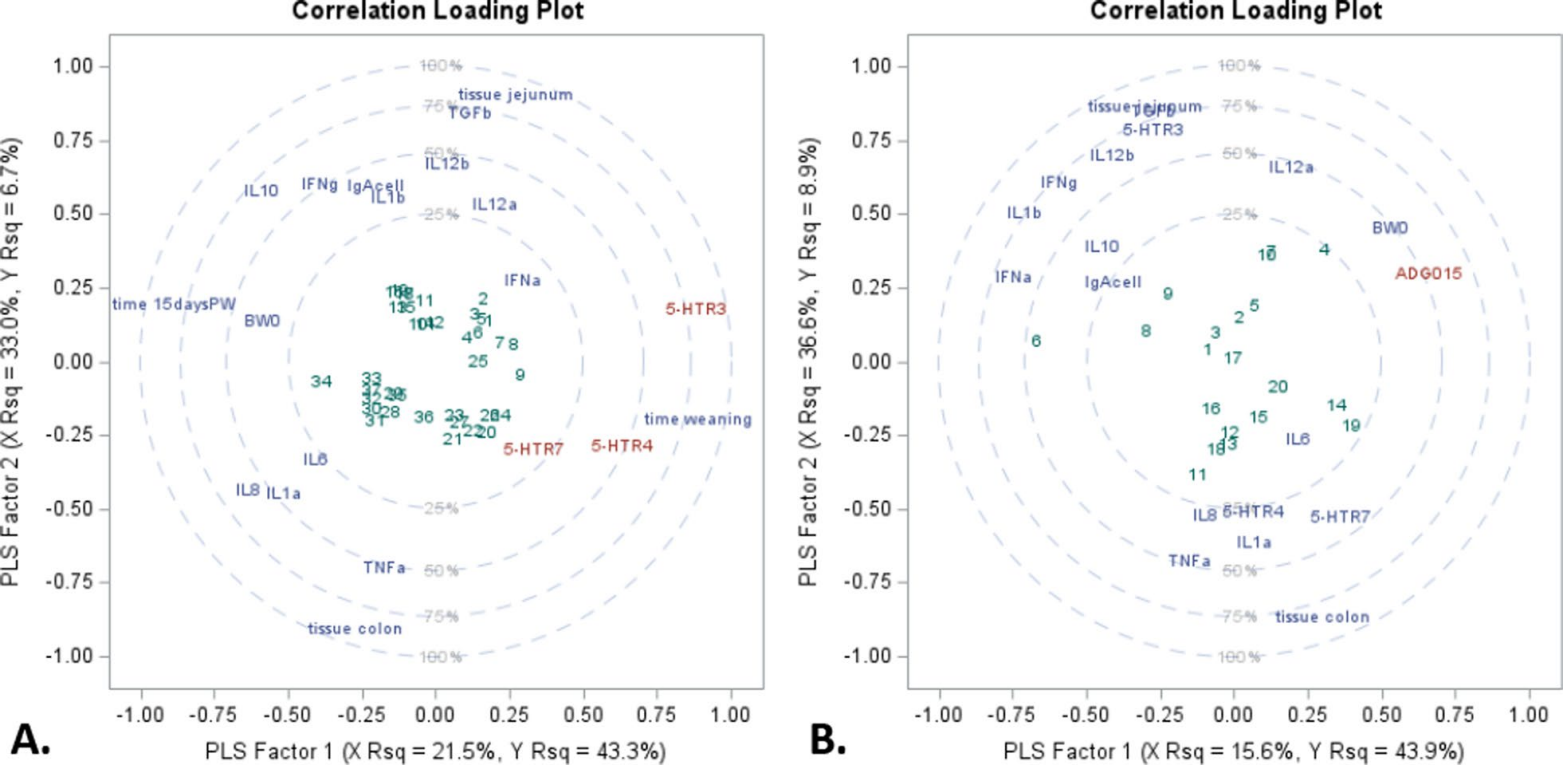
Partial least square regression (PLS) and two extracted factors for **A** dataset-C and **B** subset of 15 days post-weaning of dataset-C. **A** Variance of serotonin (5-HT) receptors (5-HTR3, 5-HTR4, and 5-HTR7) explained by time (1- and 15-days post-weaning), tissue (jejunum and colon), mRNA expression of 12 immune biomarkers^1^ from jejunum and colon, and body weight at weaning (BW0) from piglets at 1 day (n = 9) and 15 days post-weaning (n = 10). **B** Variance of average daily gain from weaning to 15 days post-weaning (ADG015) explained by BW0, tissue (jejunum and colon), and mRNA expression of 12 immune biomarkers^1^ and serotonin receptors^2^ from piglets at 15 days post-weaning (n = 10). ^1^mRNA expression (log 2) of 11 cytokines: interleukin IL-1α (IL1a), IL-1β (IL1b), IL-6 (IL6), IL-8 (IL8), IL-10 (IL10), IL-12p35 (IL12a), IL-12p40 (IL12b), tumor necrosis factor alpha (TNFa), interferon alpha and gamma (IFNa and IFNg), transforming growth factor betta (TGFb) for jejunum, colon and PBMCs; and IgA-producing cells (IgAcell) for jejunum and colon. ^2^mRNA expression (log 2) of serotonin (5-HT) receptors (HTR3, HTR4, and HTR7)

The correlation loading plot of the PLS regression analysis (Fig. 3), consists of overlaid scatter plots of the scores (green) of the first two factors, the loadings of the model effects (blue), and the loadings of the dependent variables (red). The loadings are scaled so that the amount of variation in the variables that is explained by the model is proportional to the distance from the origin, overlaid with circles depicting the levels of explained variation. PLS analysis conducted using dataset-C (see “Statistical analysis” section) reveals that 5-HTR3 expression accounts for 76% of the variability in the three-receptor model (Fig. 3A). Receptor expression is explained primarily by time (x axis as Factor 1), and by time of weaning and tissue (y axis as Factor 2), which are then organized as the scores in 4 concentric circles (clouds; Fig. 3). Immune parameter responses also partly explain 5-HTR receptor variance and appear clustered by time, tissue, or the combination thereof. More specifically, TGF-β, IL-12β, and IL-12α cluster with the jejunum, whereas TNF-α is grouped with the colon. Cytokines IL-8, IL-6 and IL-1α are grouped with colon and 15 days post-weaning, whereas IL-10, IFN-γ, IL-1β and IgA-producing cells are clustered with jejunum tissue and 15 days post-weaning.

PLS analysis was also conducted using a subset from dataset-C (Fig. 3B) to determine which factors account for the variability in ADG from weaning to 15 days post-weaning. Weaning BW (start weight) was most closely associated with ADG, while cytokine groups were split by tissue types. The grouping of cytokines by tissue, jejunum, or colon, appears to be similar in PLS analysis conducted for 5-HT receptor expression (Fig. 3A) and ADG (Fig. 3B). More specifically, those factors associated with the jejunum, from stronger to weaker correlation, include 5-HTR3, TGF-β, IL-12β, IFN-γ, IL-1β, IL-10, IL12α, IFN-α, and IgA-producing cells, whereas colon tissue was more closely associated with 5-HTR4, 5-HTR-7, TNF-α, IL-8, IL-1α and IL-6 (Fig. 3).

## Discussion

Receptors 5-HTR3 and 5-HTR4 play key roles on secretory, motility, and immunity functions in the GI tract [223] and may also be involved in intestinal homeostasis early post-weaning. These receptors were highly expressed at weaning in both jejunum and colon tissues relative to 15 days post-weaning, in agreement with observations made by Carlson et al. [21, 22]. Using an ex vivo porcine intestinal model, the authors demonstrate an enhanced chloride secretion at weaning compared to 15 days post-weaning, providing evidence of increased sensitivity to 5-HT. The increased sensitivity could be related to 5-HTR3 and 5-HTR4 receptors, which were found highly expressed in the present study and are well known for their secretory functions [1, 23]. Little is known about change in 5-HT system during the weaning process in pigs. The higher 5-HT expression at weaning relative to 15 days could be explained by emerging serotonergic mechanisms, hypothesized to influence the weaning process [24]. Otherwise, it could be related to ECs cells adapting towards interactions with new gut microbial metabolites [25] as diet changes.

Correlation analysis indicated that 5-HT receptors were associated with several immune system biomarkers at both 1- and 15-days post-weaning, while multivariate analysis also reveals strong effects of tissue and time. More correlations were found at 15 days post-weaning compared to 1-day post-weaning, while in general, receptors were expressed at a higher level at 1-day post-weaning. Our hypothesis that transcriptional upregulation of 5-HT receptors at weaning is linked to increased cytokine mRNA abundance as a cause-effect mechanism cannot be confirmed by the present data.

A limitation in the current study is the lack of a clear pre-weaning status. The piglets from the 1-day post-weaning sub-group were exposed to weaning for 18 h prior to sampling and may not strictly be considered an adequate baseline control, nor reflect complete weaning stress. In pigs, villus morphometric changes appear at day 2 post-weaning but not at day 1 [26]. Local immune activation, including IL-1β, IL-6, and TNF-α mRNA, has been shown to increase between day 0 and 2 post-weaning [27] while CD4+ and CD8+ cells (in crypts and villus) decrease from day 2 post-weaning [26]. In the neonatal piglet, the epithelial turnover rate is approximately 7 days while in the mature gut, it is 2 to 3 days [28]. The implications for the current study are that receptor expression changes may not be fully captured at 18 h post-weaning. The assessment of immunological consequences and 5-HT signaling early post-weaning may require a specific design with more targeted sampling times.

There was a lack of correlation for 5-HT receptors between the jejunum and colon regardless of timepoint and receptor. This may be explained by the differences in motility, secretory and immunity function needs between the jejunum and colon [29, 30]. ECs storing 5-HT have previously been shown to have different activation mechanisms depending on their location in the GIT. Lund et al. [25] report that the release of 5-HT from ECs in the small intestine is strongly regulated by a paracrine mechanism involving glucagon-like peptide-1 (GLP-1) and secondary to neighboring enteroendocrine cell signaling. Conversely, colonic ECs express a large range of receptors and sense chemical stimuli directly from the lumen [25].

Cytokine signaling can initiate a pro- or anti-inflammatory response [31]. In the present study, determination of the correlation of 5-HT receptor with cytokine mRNA abundance suggests an association of the 5-HT receptors with both pro- and anti-inflammatory pathway activation. Associations were mostly negative for 5-HTR3 and 5-HTR4, while 5-HTR7 showed both positive and negative correlations with cytokine mRNA. Regmi et al. [32] reported that in mice, 5-HT increases inflammatory cytokines that disrupt the epithelial cell wall and regulates innate immunity in the colon via NADPH oxidase 2-derived reactive oxygen species. Similarly, human monocytes stimulated with both lipopolysaccharides (LPS) and 5-HT increased cytokine production via action on 5-HTR3, 5-HTR4 and 5-HTR7 receptors [23]. Specifically, 5-HTR3 up-regulated the LPS-induced production of IL-1β, IL-6 and IL-8, while it did not modify TNF-α and IL-12β secretion. Receptor 5-HTR7 has been associated with a pro-inflammatory response and to pathological conditions [33], but in the context of the current study and the absence of pathology, 5-HTR7 signaling was likely related to the weaning event. Correlations for 5-HTR7 mRNA abundance at day 15 post-weaning differed between tissues. In the jejunum, 5-HTR7 expression was lower and was positively associated with pro-inflammatory biomarkers (IFN-α, IL-1α, IL-1β, IL-8, TNF-α and IL-12α). Concurrently, 5-HTR7 expression in the colon was expressed at higher levels and was negatively associated with both pro-inflammatory and anti-inflammatory biomarkers (IL-1α, IL-1β, IL-10 and TGF-β). Previous studies evaluating 5-HT effects on dendritic cells also describe contrasting roles. Shajib and Khan [4] observed pro-inflammatory effects of 5-HTR7 activation, while Idzko et al. [34] showed anti-inflammatory effects. In the current study, the three 5-HT receptors were associated with local pro-inflammatory signaling with different degrees of expression, although a relatively higher expression pattern appears to be associated with both pro-inflammatory and anti-inflammatory signaling.

Only few correlations between local expression of 5-HT receptors with adaptative and systemic immunity markers were observed. However, 5-HTR4 may play a role in systemic adaptative immunity 15 days post-weaning, as expression in the jejunum was negatively associated with lymphocytes TCD3, and positively associated with lymphocyte B. In colon, 5-HTR4 was negatively correlated with local IgA-producing cells, but positively correlated with systemic Tc and CD4+ CD8+, which could suggest a potential role in T cell differentiation and function. Various roles of the 5-HT system on adaptative immunity have been described [4, 12, 26], although more research is warranted in piglets.

At weaning, villus height was positively associated with increased expression of 5-HTR4. Crypt depth was positively correlated with 5-HTR7, while villus height to crypt depth ratio was positively associated to both 5-HTR3 and 5-HTR4. These findings suggest that morphometric traits in a healthy epithelium [35] at 1-day post-weaning may be associated with 5-HTR3 and 5-HTR4 receptor gene expression. One explanation could be that villus length, and hence surface area, by quantity of receptors, includes a greater amount of mRNA. However, this contrasts with the lack of association between 5-HTR expression and morphometry at 15 days post-weaning, suggesting a relationship with the epithelium phenotype and health. The weaning process-associated stressors in pigs are known to damage the intestinal epithelium and cause inflammation [36–38]. The damage reduces expression of key proteins for nutrient absorption (i.e. Golgi vesicle transport) [39]. It can be speculated that variance in short-term changes after 1-day post-weaning, such as the shortening of villus, loss of some epithelial cells and ECs, etc., may have effects on the GI 5-HT system, including the loss of receptors, 5-HT secretory imbalance or compromised 5-HT reuptake.

Beumer et al. [40] described an uneven distribution of ECs linage throughout crypt to villus axis in an organoid model. Furthermore, *lamina propia* myenteric neurons, Paneth cells and crypt surroundings more densely express 5-HT receptors than enterocytes and villus axis [41–43]. Different degrees of villus damage may influence 5-HT receptor expression in the *lamina propia* and crypts or reduce the amounts of ECs and 5-HT receptors on villus axes. Alternatively, stress factors may imbalance 5-HT release and reuptake, causing inflammation. According to Kojima et al. [44], the 5-HT released by damaged ECs during an allergic reaction intensifies peristalsis, contributing to diarrhea. 5-HT is synthesized by ECs and secreted both into the lumen and interstitial space and is thereafter removed by the serotonin-selective reuptake transporter SERT by epithelial cells [45]. It is likely that 5-HT uptake is compromised due to epithelial damage and inflammation. In fact, it has been reported that inflammatory processes led to the downregulation of SERT in a mouse model [46]. Previous authors have suggested that during stress-induced diarrhea, blocking the reuptake of 5-HT might intensify the stress condition, inducing a pathological state in the intestine [47]. This would not occur under a healthy status because 5-HT activity is terminated by SERT [45]. Feeding behavior may also be compromised by endocrine factors in response to 5-HT receptor overstimulation. The interaction between 5-HT and GLP-1 systems plays a role with hypophagia, as the associations between anorectic and noxious/stressful stimuli, which was recently demonstrated in rats [48]. Hence, we suggest that future investigations of 5-HT system in piglets should consider the inclusion of intestinal GLP-1 and SERT in their evaluation. Unfortunately, the relatively narrow scope of data herein and lack of literature on post-weaning stress in piglets limits further discussion, although results of this study offer sufficient evidence to suggest a 5-HT imbalance occurring through the transition from 1 to 15 days post-weaning.

## Conclusions

The present exploratory study provides the first in vivo evidence of the 5-HT receptor family transcriptional response and their associations with the immune system and intestinal morphometry in piglets during the post-weaning period. The 5-HT receptors were highly expressed at weaning in both the jejunum and colon tissues relative to 15 days post-weaning. Although a clear relationship between immune system and 5-HTR expression is observed, especially at day 15, a cause-consequence effect cannot be proven with the current data. Some limitations, including the lack of pre-weaning status, require attention and further research is warranted. Given the well-known roles of the 5-HT system in other species, including implications in disease states, the present study highlights the need for further research to elucidate the interaction with GI inflammation and homeostasis in weaning piglets. Such research could be the basis for new treatments or preventative interventions to ease weaning stress and reduce post-weaning diarrhea.

## Materials and methods

### Animals, data, and design

The present preliminary study uses data and samples from a larger scale study from de Groot et al. (manuscript submitted). The objective of the larger study was to evaluate immune modulation response and intestinal morphometry as induced by a novel feed additive (blend of mannan-rich hydrolyzed copra meal and rye fermented with *A. subrufescens* fungi). For the present study, only control piglets were used to include a total of 19 piglets (Large White), which were selected for similar post-weaning BW (5.5 ± 0.93 kg; weaned at 22 day of age) and shared conditions as described below. The experiment was conducted at University of Murcia (UMU, Spain) in the Swine Production Unit at the Veterinary Teaching Farm of the UMU. Pigs were allocated to a pen with 9–10 piglets each. The pens (0.61 × 1.2 m) were plastic full-slated with access to one nipple drinker and one 4 space-feeder. Piglets were provided feed and water ad libitum*,* and performance was monitored.

The study included 9 piglets at 1-day post-weaning (weaning group; 5.0 ± 0.54 kg BW at weaning) and 10 piglets at day 15 post-weaning (15 days post-weaning group; 5.9 ± 1.02 kg BW at weaning). The reasoning to compare 1-day and 15-day post-weaning piglets was to describe 5-HTR under a contrasting status regarding immunity and gastrointestinal physiology, which is adapting to a new feed condition and environment by 15 days post-weaning. Piglets were weaned in the afternoon and transported to the nursery barn where they had free access to control feed and water manufactured at a local feed mill (Pigalomar, Spain). Piglets were humanly euthanatized via an intravenous overdose (50 mg/kg BW) of barbiturate thiobarbital (Braun Medical S.A., Spain) for sample collection either the following morning (18 h after weaning) the weaning group or at day 15-post weaning in the morning. Piglets from the 15 days post-weaning group were routinely vaccinated against porcine circovirus-2 (Porcilis PCV-2, MSD, The Netherlands) at day 4 post-weaning. The commercial diet (based on 30.0% barley, 24.0% wheat, 17.2% corn, 6.0% soybean meal, 6.0% milk protein, 5.0% whey powder) had nutritional levels of Zn (ie. no pharmacological levels of ZnO) and did not contain antibiotics and were formulated to be at or above NRC requirements for pigs (NRC, 2012): 2,560 kcal/Kg net energy, 17% crude protein and 1.15% standard ileal digestible lysine.

### Blood and tissue sampling

Samples included blood and jejunum tissue and colon tissue at both 1- and 15-days post-weaning. Prior to euthanasia, 10 mL blood was collected from vena jugular into ethylenediaminetetraacetic acid (EDTA) tubes (Vacutainer, Becton Dickinson, UK) from each piglet and preserved in RNAlater. The PBMCs were isolated by Ficoll Histopaque gradient and preserved in RNAlater (Life Technologies, USA) at − 80 °C after 24 h of refrigeration at 8 °C. Blood tubes were centrifuged (10 min, 251 relative centrifugal force at room temperature) to separate serum and plasma and preserved at − 80 °C until analysis. After euthanasia, two 5 cm tissue samples were obtained from the middle section of jejunum (3.5 m distal to the ligament of Treitz) and apex section from the spiral colon (cm from ileocecal ostium not measured). Two whole tissue samples per tissue were collected and prepared as complete trans-sectional part of the intestine. The rationale for selecting these tissues was to characterize the 5-HT receptors in jejunum and colon, as they have differences in motility, secretory and immunity functions, while ECs have different activation mechanisms in each tissue [25]. Tissue samples were gently rinsed in water and a 40 mg sample was preserved in RNAlater (Life Technologies, USA), and the remainder was fixed in 10% buffered formaldehyde.

### Jejunum morphometry

A total of five photomicrographs from prepared jejunal tissue histology sections were taken at 10× magnification mounted on a glass slide and read under a Zeiss Axiocam 503 color camera (Carl Zeiss, Oberkochen, Germany) on a Zeiss Axioskop 40 microscope (Carl Zeiss, Oberkochen, Germany). Villus height (axis top to villous-crypt junction) and crypt depth (from villus-crypt junction to the base of villus) were measured with the ZEISS Efficient Navigation software (Carl Zeiss, Oberkochen, Germany). For the assessment, ten randomly selected well-oriented intact villus and crypts were measured per piglet and tissue to calculate mean villus height and crypt depth. Additionally, the villus to crypt ratio was calculated. All morphometric measurements were performed by the same researcher who was blinded to treatments and timepoints.

### Immunohistochemistry for IgA-producing cells

For detection of IgA-producing cells in the jejunum and colon tissue, the avidin–biotin–peroxidase complex technique was used. The samples were embedded in paraffin-wax to obtain 5 μm thick sections. The samples were then dewaxed and dehydrated with graded ethanol and the endogenous peroxidase activity was quenched in 3% H_2_O_2_ in methanol for 30 min. Samples were pretreated with 10% pronase in TBS (Sigma-Aldrich, USA) for antigen retrieval (12 min). Subsequently, the samples were rinsed 3 times × 5 min in TBS and incubated for 30 min in a humid chamber (20 °C) with 100 μL of blocking solution per slide. The samples were then incubated for 1 h at 37 °C with the primary antibody (goat–anti-pig IgA, Bethyl, USA) diluted 1:3000 in TBS. The secondary antibody (biotin conjugate rabbit anti-goat, Dako, USA), diluted 1:300 in TBS, was incubated for 30 min at 20 °C. The Vectastain^®^ Elite ABC kit (Vector, USA) was applied for 1 h at 20 °C. Positive labeling was detected using 3,3′-diaminobenzidine tetrahydrochloride (Dako, USA). Sections were counterstained with Mayer's haematoxylin, dehydrated and mounted. The number of IgA-producing cells in the intestinal lamina propria was counted using a Zeiss Axioskop 40 microscope (Carl Zeiss, Oberkochen, Germany) with a Spot Insight camera and the Spot Advanced software (Spot Imaging Solution, Michigan, USA). Ten non-overlapping and consecutive high magnification fields were used for counting immunolabeled cells which were expressed as amount of cells/25.000 μm^2^.

### Lymphocytes B, T and NK

The subpopulations of lymphocytes B (phenotype CD21+), NK (phenotype CD3−16+56+) and T phenotypes CD3+, T helper CD3+CD4+ (Th), T cytotoxic CD3+CD8+ (Tc), CD4+CD8+, CD4−CD8−, were analyzed by flow cytometry at ^“^Instituto de Inmunología Clínica y Enfermedades Infecciosas” (Málaga, Spain) in blood. A fresh 50 µL sample of peripheral blood per piglet was prepared for cytometry and mixed with antibody cocktail according to the manufacturer. Commercial antibodies were used separately for each tube including mouse anti-pig CD16:RPE and mouse anti-pig CD45:FITC from Bio-RAD (CA, USA), mouse anti-pig CD56:RPE from BioLegend (CA, USA), PerCP-Cy5.5 mouse anti-pig CD4a, mouse anti-pig CD8a, PE-Cy7 mouse anti-pig CD3e, and PE-Cy5 mouse anti-human CD21 from BD Pharmingen^MT^ (Becton, Dickinson and Company, NJ, USA). After incubation, samples were lysed with a commercial lyse (FACS LYSING, Becton, Dickinson and Company, NJ, USA). Subsequently, the preparations were analyzed in the FC500 cytometer (Beckman Coulter, IN, USA) at 488 nm (blue) and obtaining analysis matrices for each analyte.

### Gene expression for serotonin receptors and cytokines

The 5-HT receptors transcriptional response in jejunum and colon tissues were determined by means of relative quantification, using three primer pairs (Table 2) designed using Primer Blast^®^ (National Center for Biotechnology Information, Bethesda, MD, USA) from sequences XM_003357301.4, NM_001001267.1 and NM_214085.1 for receptors 3, 4 and 7, respectively. Gene expression for cytokines IL-1α, IL-1β, IL-6, IL-8, IL-10, IL-12p35 (IL-12α), IL-12p40 (IL-12β), TNF-α, IFN-α and IFN-γ and TGF-β were determined by means of relative quantification, using primers previously described (Table 2). Samples included jejunum, colon and PBMCs extracted from blood samples. Total RNA was isolated from 20 mg of tissue samples, and from PBMC by using the Micro RNeasy kit (Qiagen, USA) and DNAc was synthetized using the Geneamp RNA PCR Core Kit (Life Technology, USA) using oligo-dT as primers to get cDNA only from mRNA. The PCRs were performed using a 7300 ABI thermocycler (Life Technologies, USA) and the GoTaq^®^ qPCR Master Mix (Promega, USA) with SYBR-Green chemistry. The specificity of the reaction was assessed by analyzing the melting curve. The efficiency of qPCR was between 91 and 105% and the correlation coefficient for the standard curves was minimum 0.972. The samples were normalized using the average Ct for glyceraldehyde-3-phosphatedehydrogenase (GAPDH), cyclophilin and β-actin. The expression for each sample was calculated by Pfaffl’s method [49].

**Table 2:** Primers for serotonin (5-HT) receptors 3, 4, and 7 designed^1^, cytokines^2^ and housekeeper genes^3^

	Primer forward (5′ → 3′)	Primer reverse (5′ → 3′)	References
5-HTR3	5′-AACTACAAGCCCCTCCAGGT-3′	5′-CATGAAAACACTCCTGTCGAA-3′	This paper
5-HTR4	5′-CGTCTGGATTTATGGGGAGA-3′	5′-GAACAAGATGACCCCTCTGC-3′	This paper
5-HTR5	5′-GCCACTTCTTCTGCAACGTC-3′	5′-CAAGCACACCTTGTCGTCAT-3′	This paper
IL-1α	5′-GTGCTCAAAACGAAGACGAACC-3′	5′-CATATTGCCATGCTTTTCCCAGAA-3′	[50]
IL-1β	5′-AACGTGCAGTCTATGGAGT-3′	5′-GAACACCACTTCTCTCTTCA-3′	[51]
IL-6	5′-CTGGCAGAAAACAACCTGAACC-3′	5′-TGATTCTCATCAAGCAGGTCTCC-3′	[51]
IL-8	5′-GCTCTCTGTGAGGCTGCAGTTC-3′	5′-AAGGTGTGGAATGCGTATTTATGC-3′	[52]
IL-10	5′-TGAGAACAGCTGCATCCACTTC-3′	5′-TCTGGTCCTTCGTTTGAAAGAAA-3′	[53]
IL-12p35	5′-AGTTCCAGGCCATGAATGCA-3′	5′-TGGCACAGTCTCACTGTTGA-3′	[53]
IL-12p40	5′-TTTCAGACCCGACGAACTCT-3′	5′-CATTGGGGTACCAGTCCAAC-3′	[54]
TNF-α	5′-ACTCGGAACCTCATGGACAG-3′	5′-AGGGGTGAGTCAGTGTGACC-3′	[55]
IFN-α	5′-CCCCTGTGCCTGGGAGAT-3′	5′-AGGTTTCTGGAGGAAGAGAAGGA-3′	[56]
IFN-γ	5′-TGGTAGCTCTGGGAAACTGAATG-3′	5′-GGCTTTGCGCTGGATCTG-3′	[53]
TGF-β	5′-CACGTGGAGCTATACCAGAA-3′	5′-TCCGGTGACATCAAAGGACA-3′	[56]
β-actin	5′-CTACGTCGCCCTGGACTTC-3′	5′-GATGCCGCAGGATTCCAT-3′	[57]
Cyclophilin	5′-TGCTTTCACAGAATAATTCCAGGATTTA-3′	5′-GACTTGCCACCAGTGCCATTA-3′	[58]
GAPDH	5′-ACATGGCCTCCAAGGAGTAAGA-3′	5′-GATCGAGTTGGGGCTGTGACT-3′	[58]

^1^Designed using Primer Blast^®^ (Bethesda, MD, USA) from sequences XM_003357301.4, NM_001001267.1 and NM_214085.1

^2^Cytokines = interleukin (IL)-1α, IL1-β, IL-6, IL-8, IL-10, IL-12p35 (IL-12α), IL-12p40 (IL12-β), tumor necrosis factor alpha (TNF-α), interferon alpha and gamma (IFN-α and IFN-γ), transforming growth factor betta (TGF-β)

^3^β-actin, cyclophilin and glyceraldehyde-3-phosphatedehydrogenase (GAPDH)

### Statistical analysis

All data were processed using SAS (version 9.4, SAS Institute; Cary, USA). Gene expression data were transformed to log2 for normalization. Two datasets were built to assess current hypotheses. Dataset-A included all tissues (jejunum, colon and PBMCs) but had the analytical weakness of not including: (1) 5-HT receptors on PBMCs, and (2) immune cells on jejunum and colon. Dataset-C was built to be complete with all variables of interest and GI tract (jejunum and colon samples). Dataset-C included 5-HT receptor mRNA expression (5-HTR3, 5-HTR4, and 5-HTR7) and immune parameters (mRNA gene expression of 11 cytokines and quantify of IgA-producing cells) which were nested within timepoint and tissue.

Dataset-C was analyzed by MIXED model procedure (PROC MIXED) from SAS with time, group (weaning and day 15 post-weaning) and tissue (jejunum and colon) as main effects, and tissue being included as a repeated measure. Treatment means were separated by using the LSMEANS statement, PDIFF option and SIMULATE adjustment for multiple comparisons. Individual pig was used as the experimental unit. *P* values < 0.05 were considered significant and *P* values < 0.10 were considered a trend.

Dataset-A, was analyzed using Pearson correlation coefficients to measure the relation between gene mRNA expression of 5-HTR3, 5-HTR4, and 5-HTR7 receptors on intestinal tissue (jejunum and colon) with receptors themselves, weaning BW, mRNA gene expression of 11 cytokines in jejunum, colon and PBMCs, quantity of IgA-producing cells in jejunum and colon, morphometry traits (villus height, crypt depth and ratio) in tissue jejunum, and immune cells cytometry from blood.

Multivariate analyses were performed using partial least squares regression (PLS) and a principal component analysis (PCA, with multidimensional scaling and orthogonal transformation for rotation). The PCA was applied first to dataset-A (all parameters) including the variables serotonin receptors, mRNA gene expression of 11 cytokines and IgA-producing cells quantity coded as jejunum, colon or PBMC, 7 immune cell types from blood, jejunum villus height and crypt depth and weaning body weight (BW0). variables weaning BW, mRNA gene expression of 11 cytokines and IgA-producing cells quantity in the two intestinal tissues with tissue and time as nesting effects. The PCA procedure was repeated on dataset-C this time including only serotonin receptors, BW at weaning, and mRNA gene expression of 11 cytokines and IgA-producing cells quantity in the two intestinal tissues with tissue and time as nesting effects. Because dataset-A was incomplete, time and tissue could not be nested effects for immune variables and each sample pertained to a different time and tissue.

The PLS analysis was set to assess: (1) variance of serotonin receptors explained by time (1 and 15 days post-weaning), tissue (jejunum and colon), and immune biomarkers from dataset-C; (2) variance of serotonin receptors explained by all immune biomarkers for each tissue (jejunum, colon and PBMCs) and time (1 day and 15 days post-weaning) separately using dataset-A (data not shown as PLS model extracted only 1 factor which explained only 14% of variance); (3) variance of ADG for 15 days post-weaning explained by BW at day 1, 5-HT receptors tissue (jejunum and colon), and immune biomarkers as subset from dataset-C; (4) variance of ADG for 15 days post-weaning (dataset-C subset) explained only by weaning BW and by 5-HT receptors with tissue effect (jejunum and colon); (5) variance of ADG for 15 days post-weaning explained by weaning BW, 5-HT receptors and all immune biomarkers for each tissue (jejunum, colon and PBMCs) and time (1 and 15 days post-weaning) separately as subset of dataset-A (data not shown as PLS did not converge).

## Data Availability

The datasets generated and/or analysed during the current study are not publicly available due ownership by Nutreco N. V. but are available from the corresponding author on reasonable request.
